# Particle-Induced Pulmonary Acute Phase Response Correlates with Neutrophil Influx Linking Inhaled Particles and Cardiovascular Risk

**DOI:** 10.1371/journal.pone.0069020

**Published:** 2013-07-24

**Authors:** Anne Thoustrup Saber, Jacob Stuart Lamson, Nicklas Raun Jacobsen, Gitte Ravn-Haren, Karin Sørig Hougaard, Allen Njimeri Nyendi, Pia Wahlberg, Anne Mette Madsen, Petra Jackson, Håkan Wallin, Ulla Vogel

**Affiliations:** 1 The National Research Centre for the Working Environment, Copenhagen, Denmark; 2 National Food Institute, Technical University of Denmark, Søborg, Denmark; 3 Danish Technological Institute, Taastrup, Denmark; 4 Institute of Public Health, University of Copenhagen, Copenhagen, Denmark; 5 Department of Micro- and Nanotechnology, Technical University of Denmark, Lyngby, Denmark; University of Giessen Lung Center, Germany

## Abstract

**Background:**

Particulate air pollution is associated with cardiovascular disease. Acute phase response is causally linked to cardiovascular disease. Here, we propose that particle-induced pulmonary acute phase response provides an underlying mechanism for particle-induced cardiovascular risk.

**Methods:**

We analysed the mRNA expression of *Serum Amyloid A* (*Saa3*) in lung tissue from female C57BL/6J mice exposed to different particles including nanomaterials (carbon black and titanium dioxide nanoparticles, multi- and single walled carbon nanotubes), diesel exhaust particles and airborne dust collected at a biofuel plant. Mice were exposed to single or multiple doses of particles by inhalation or intratracheal instillation and pulmonary mRNA expression of *Saa3* was determined at different time points of up to 4 weeks after exposure. Also hepatic mRNA expression of *Saa3*, SAA3 protein levels in broncheoalveolar lavage fluid and in plasma and high density lipoprotein levels in plasma were determined in mice exposed to multiwalled carbon nanotubes.

**Results:**

Pulmonary exposure to particles strongly increased *Saa3* mRNA levels in lung tissue and elevated SAA3 protein levels in broncheoalveolar lavage fluid and plasma, whereas hepatic *Saa3* levels were much less affected. Pulmonary *Saa3* expression correlated with the number of neutrophils in BAL across different dosing regimens, doses and time points.

**Conclusions:**

Pulmonary acute phase response may constitute a direct link between particle inhalation and risk of cardiovascular disease. We propose that the particle-induced pulmonary acute phase response may predict risk for cardiovascular disease.

## Introduction

Inhalation of particles by air pollution, smoking and occupational exposure cause pulmonary inflammation and are risk factors for cardiovascular disease [1], [2]. The mechanisms by which particles induce cardiovascular diseases are not well understood. It is a generally held view that the particle-induced pulmonary inflammation leads to release of cytokines into the circulation that triggers a liver-mediated acute phase response which, in turn, promotes cardiovascular disease [2]–[5].

The acute phase response is a systemic response to acute and chronic inflammatory states caused by a variety of factors including bacterial infections, trauma, and infarction [6]. Acute phase response and the accompanying inflammatory response are strongly associated to increased risk of cardiovascular disease in epidemiological studies [7]–[9]. For example periodontal pathogen infection and virus infections are associated with risk of cardiovascular disease [10], [11]. Blood levels of the acute phase proteins C-Reactive Protein (CRP) and Serum Amyloid A (SAA) are among the strongest known risk factors for cardiovascular diseases in prospective studies [12]. An association between air pollution and CRP levels has been observed in large cross-sectional and prospective studies [13], [14]. This indicates that particle-induced inflammation and acute phase response may be important for cardiovascular disease.

The acute phase response is characterised by up- and down regulation of blood levels of a variety of proteins, termed acute phase proteins, such as CRP, SAA and fibrinogen [6]. In mice, *Saa3* is expressed in various tissues including lung and liver whereas *Saa1* and *Saa2* are considered liver specific [15]. In humans, *SAA3* is a pseudogene [16] and *SAA1* and *SAA2* are expressed both hepatically and extra-hepatically [17]. CRP is expressed to a very limited extent in mice [18].

We have previously demonstrated that inhalation and instillation of the carbon black nanoparticle Printex 90 (NanoCB), and the titanium dioxide nanoparticle UV-Titan L181 (NanoTiO_2_) induce long-lasting pulmonary inflammation [19]–[23]. Unexpectedly, this was not accompanied by a hepatic acute phase response [19], [24]: In contrast, both pulmonary exposure to NanoCB and NanoTiO_2_ induced a strong pulmonary acute phase response [19], [25], [26]. Thus, instillation of NanoCB lead to increased gene expression of several acute phase genes including *Saa3, Saa2, Saa1*, *Metallothionein 2 (Mt2), Ceruloplasmin (Cp), Metallothionein 1 (Mt1), Orosomucoid 2 (Orm2), Orosomucoid 1 (Orm1),* and *Complement component 3 (C3)* 24 hours after exposure [25]. Pulmonary exposure to NanoTiO_2_ lead to increased expression of 44 acute phase genes including *Saa1, Saa2, Saa3,C3, Il1b, C-C motif chemokine 4 (Ccl4), C-C motif chemokine 17 (Ccl17), Chemokine (C-X-C motif) ligand 5*.

*(Cxcl5), S100 calcium-binding protein A8 (S100a8), S100 calcium binding protein A9 (S100A9)*, and NF-kappa-B inhibitor alpha (*Nfkbia)* 24 hours after exposure with dose-dependency in both the number of differentially expressed acute phase genes and the observed expression fold [26]. For both particle exposures, *Saa3* was the most differentially expressed gene in murine lung tissue with 65-fold increase following NanoCB deposition [25] and up to 100-fold increase following NanoTiO_2_ deposition [26], one day after intratracheal instillation of 162 µg of particles. Thus, we here use pulmonary *Saa3* gene expression in lung tissue as a biomarker of pulmonary acute phase response because of the large dynamic range in *Saa3* gene expression.

We hypothesize that pulmonary deposition of particles in the lung triggers long-lasting pulmonary induction of acute phase proteins including *Saa3* followed by lung secretion of acute phase proteins including SAA3 into blood. We propose that pulmonary acute phase response may constitute a direct link between particle inhalation and risk of cardiovascular disease and that air pollution-induced cardiovascular disease may thus be a direct consequence of pulmonary secretion of acute phase proteins that are known to affect many aspects of homeostasis such as plaque progression [27]–[29] and endothelial function [30].

## Methods

### Ethics Statement

The experiments were approved by the Danish “Animal Experiments Inspectorate” and carried out following their guidelines for ethical conduct and care when using animals in research.

### Mice

C57BL/CJ female mice M&B or BomTac from later Taconic Denmark were used.

The virgin mice were allowed to acclimatize for 2 weeks and the time-mated mice were received on gestation day 3 (day of plug GD1). All mice were given food (Altromin 1324) and water ad libitum. The mice were group housed in polypropylene cages with sawdust bedding and enrichment (removed during nursing) at controlled temperature 21±1°C and humidity 50±10% with a 12-h light:12-h dark cycle.

### Particles

The following materials were used in this study: carbon black (NanoCB), titanium dioxide (NanoTiO_2_), diesel exhaust particles (DEP), multiwalled carbon nanotubes (MWCNT), single-walled carbon nanotubes (SWCNT_1_ and SWCNT_2_) and airborne dust collected at a biofuel facility (boiler room and straw storage). The NanoCB, Printex 90 was a gift from Degussa-Hüls, Germany. The NanoTiO_2_, UV-Titan L181 (Kemira Pigments, Finland) was a gift from Boesens Fabrikker Aps, Denmark. The DEP was a standard reference material (SRM) 2975 purchased from the National Institute of Standards and Technology, USA. The MWCNT was a gift from Mitsui, Japan. The SWCNT_1_ was purchased from Sigma, USA. The SWCNT_2_ was purchased from Thomas Swan, USA. Airborne dust was collected from a boiler room (Boiler dust) and a straw storage hall (Straw dust) at a biofuel plant as described previously [31].

### Exposure of mice

The study consists of six parts. An overview of the studies is given in Table 1. Study 1 is part of a larger set-up in which mice were given a single intratracheal instillation of a range of different types of particles. We have previously reported inflammatory and DNA damaging effects in mice given NanoTiO_2_ [20], [26] and NanoCB [20], [21] from this set-up.

**Table 1:** Overview of studies and nanomaterials.

Study	Exposure way	Particles	Deposited dose (µg)	Declared particle size	Agglomerated size in inhalation aerosol or instillation suspension	Reference
1	Intratracheal	NanoCB	18, 54, 162	14 nm	200 nm^?^	[20]
	instillation	NanoTiO2	18, 54, 162	17 nm	100 nm^?^	
		MWCNT	18, 54, 162	40–50 nm×1–4 µm	ND^§^	
		SWCNT_1_	18, 54, 162	1.1 nm×0.5–100µm	ND^§^	
		SWCNT_2_	18, 54, 162	0.8–1.7nm×≤1 µm	ND^§^	
2^**^	Intratracheal	MWCNT	18, 54, 128	40–50 nm×1– 4 µm	ND^§^	
	instillation					
3	Inhalation	NanoCB	75	14 nm	65 nm^‡^	[32]
		DEP	19*	1.62 µm^†^	215 nm^‡^	
						
4	Inhalation	NanoTiO_2_	73	17 nm	97 nm^‡^	[19]
5	Inhalation	NanoCB	287	14 nm	310 nm (bimodal; 290 and 1500 nm)^‡^	[22]
6	Intratracheal	Boiler dust	216	ND^§^	ND^§^	[31]
	instillation	Storage dust	216	ND^§^	ND^§^	

* Based on a deposition fraction similar to NanoTiO_2_,^†^ Mean diameter (number distribution) from National Institute of Standards and Technology, Certificate of Analysis, Standard Reference Material® 2975,^‡^ Geometric mean, ^§^ Not detetermined, ^?^ Hydrodynamic size, ^**^ Study 2 was an additional experiment performed to obtain plasma from MWCNT instilled mice and control animals.

Study 3–6 consist of experiments with mice that we have reported of previously [20], [22], [23], [31], [32], and for which lung tissue in this study was reanalyzed for *Saa3* mRNA expression.

#### Exposure by intratracheal instillation

The instillation procedure has been described in detail previously [20]. In brief, the mice were given either 18 µg, 54 µg or 162 µg of particles (corresponding to ca. 0.9, 2.8 and 8.4 mg/kg, respectively) by a single i.t. instillation or 4 multiple instillations. After exposure, tissue and BAL cells were for all studies prepared as described previously [32].

**Figure 1:**
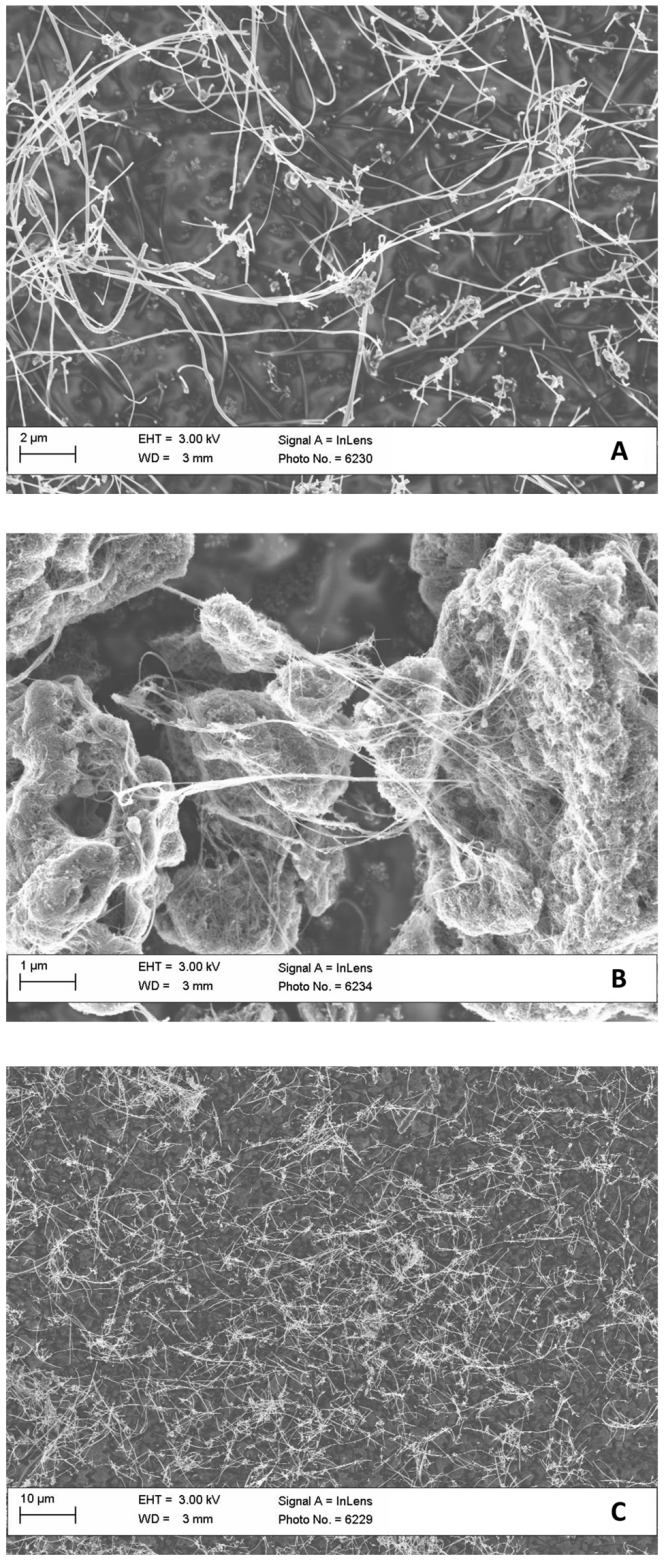
Scanning electron microscope images of CNTs on powder form. A) MWCNT; B) SWCNT_1_, and C) SWCNT_2_.

For study 1 characterization of NanoCB and NanoTiO_2_ in instillation vehicle have been published previously [20], [33]. For the rest of the particles in study 1 (MWCNT, SWCNT_1_, SWCNT_2_), the average size of the materials in instillation vehicle were analyzed by Dynamic Light Scattering (DLS) and the shapes of the materials and the extent of agglomeration/aggregation in instillation vehicle were characterized by scanning electron microscopy (SEM). Electron microscopy was performed on both pristine CNTS and CNTs in instillation vehicle. However, SEM pictures of CNTs in vehicle were dominated by salt crystals from the saline solution (results not shown). SEM pictures of the dry CNTs are shown in Figure 1. SWCNT_1_ and SWCNT_2_ both appeared bundled, hampering assessment of dimensions. This was true both when evaluated as powders and in suspension for instillation. DLS measurements of the CNTs suspensions indicated the presence of agglomerates in the µm-range probably due to bundling of CNTs and sedimentation (results not shown). A detailed characterization of the particles from study 3–6 was reported previously [20], [22], [24], [31]. Selected data are summarized in Table 1.

#### Exposure by inhalation

Mice exposed by inhalation were exposed in a 18 L nose-only exposure chamber as previously described [22], [23], [32]. In brief, the particles were aerosolized by a microfeeder with dispersion nozzle (Fraunhofer Institut für Toxikologie and Aerosolforschung, Hannover, Germany). The number of particles was measured by a condensation particle counter (TSI model 3022A). The particle concentration was measured periodically during exposure by weighing of filters.

### Set-up of studies

#### Study 1

The set-up of the single intratracheal instillation of nanomaterials has been described in detail for the NanoCB and NanoTiO_2_ [20]. The mice exposed to the three types of CNTs were exposed similarly. In brief, mice received a single intratracheal instillation of 18, 54 and 162 µg of NanoTiO_2_, NanoCB, MWCNT, SWCNT_1_ or SWCNT_2_ and were evaluated 1, 3 and 28 days after intratracheal instillation. Particles were suspended by sonication in 0.9% NaCl MilliQ water containing 10% v/v acellular BAL from C57BL/6 mice. The BAL fluid was prepared by flushing unexposed mice twice to 0.6 ml 0.9% NaCl solution yielding approximately 1 ml of BAL fluid. Acellular BAL was prepared by centrifugation of BAL fluid at 400 g (10 min, 4°C). The particles (4.05 mg/ml) were sonicated using a 400 W Branson Sonifier S-450D (Branson Ultrasonics Corp., Danbury, CT, USA) equipped with a disruptor horn (Model number: 101-147-037). Total sonication time was 16 min, with alternating 10 s pulses and 10 s pauses at amplitude of 10% (8 min sonication in total). Samples were continuously cooled on ice during the sonication procedure. Vehicle control solutions were prepared containing 90% 0.9% NaCl MilliQ water and 10% acellular BAL fluid. Lungs were snap frozen in liquid nitrogen and stored at −80°C.

#### Study 2

Because no plasma was saved from study 1 a supplementary study was performed similarly to study 1 to obtain plasma from mice intratracheally instilled with MWCNT and control vehicle. In a separate experiment, blood was collected from groups of 3 C57BL/6 mice 3 days after instillation of a single dose of 18 µg, 54 µg, or 128 µg of MWCNT. Particle suspensions were prepared as described for study 1. The reason for the different highest dose (128 µg) was that these mice also were part of another study.

#### Study 3

The design of the repeated exposure to NanoCB and DEP has been described in detail elsewhere [24]. In brief, we exposed C57 BL/6J virgin mice by inhalation to 20 mg/m^3^ DEP, NanoCB or filtered air for 90 min on four consecutive days. The particle numbers of NanoCB and DEP were ca. 8.0×10^5^/cm^3^ and 9.5×10^5^/cm^3^, respectively. One hour after the last exposure the mice were killed and lungs were snap frozen in liquid nitrogen and stored at −80°C.

#### Study 4

The design of the repeated exposure to NanoTiO_2_ has been described in detail elsewhere [23]. In brief, C57BL/6J time-mated mice were exposed by inhalation 1h/day to 42 mg/m^3^ NanoTiO_2_ on gestation days 8–18. The particle number concentration was 1.70±0.20×10^6^/cm^3^. Mice that did not give birth (non-pregnant mice) were killed 5 days after the end of exposure and lungs were snap frozen in liquid nitrogen and stored at −80°C. Dams were killed 26–27 days after the last exposure (after end of lactation) and tissue was treated as described above.

#### Study 5

The design of the repeated exposure to NanoCB has been described in detail elsewhere [22]. In brief, C57BL/6J time-mated mice were exposed by inhalation to 42 mg/m^3^ NanoCB for 1 h/day on gestation days (GD) 8–18. The particle number concentration was 4.09±0.03×10^6^/cm^3^. Mice with none or few offspring were killed 5 days after the end of exposure and lungs were snap frozen in liquid nitrogen and stored at −80°C. Dams with larger litters were killed 24–25 days after the end of exposure and tissue was treated as described previously.

#### Study 6

The design of the repeated exposure to boiler dust and storage dust has been described in detail elsewhere [31]. In brief, C57 BL/6J virgin mice were intratracheally instilled on four consecutive days with 54 µg µg of airborne dust collected at a biofuel plant in the straw storage hall or to dust collected in the boiler room (total dose 216 µg/animal). Control mice were exposed similarly to a 0.9% sodium chloride solution. Endpoints were evaluated 1 hour after last exposure. Lungs were snap frozen in liquid nitrogen and stored at −80°C.

### *Saa3* mRNA expression analysis

Hepatic and pulmonary RNA from the C57BL/6 was isolated as described previously [24]. cDNA was prepared from DNase treated RNA using TaqMan reverse transcription reagents (Applied Biosystems, USA) as recommended by the manufacturer.

*Saa3* gene expression was determined using real-time RT-PCR with 18S RNA as the reference gene as previously described [24]. In brief, each sample was run in triplicate on the ABI PRISM 7700 sequence detector (PE Biosystems, Foster City, CA, USA). For *Saa1* (Mm00656927 gi) and *Sap* (Mm00488099 g1), TaqMan pre-developed reaction kits (Applied Biosystems, USA) were used. The sequences of the *Saa3* primers and probe were: Saa3forward: 5′ GCC TGG GCT GCT AAA GTC AT 3′, Saa3reverse: 5′ TGC TCC ATG TCC CGT GAA C 3′ and Saa3probe: 5′ FAM – TCT GAA CAG CCT CTC TGG CAT CGC T– TAMRA 3′. Target genes and 18S RNA levels were quantified in separate wells. The relative expression of the target gene was calculated by the comparative method 2^−ΔCt^ [34]. The average standard deviation on triplicates was 15 %. The standard deviation on repeated measurements of the same sample (the control) in separate experiments was 25%, indicating that the day-to day variation of the assay was 25%. The probes and primers have been validated and the assay was quantitative over a 256-fold range. Messenger RNA measurements were excluded if the 18S content fell outside the range in which the PCR was found to be quantitative defined by the validation experiments. Negative controls, where RNA had not been converted to cDNA, were included in each run.

### SAA3 protein analysis

Plasma SAA3 and BALF SAA3 was measured by ELISA (Mouse Serum Amyloid A-3, Cat.#EZMSAA3-12K, Millipore) according to manufacturer's instructions.

### HDL, LDL and VLDL cholesterol concentrations

Lipoproteins were separated by density gradient ultracentrifugation for 18 h at 21°C using 150 µl of plasma according to Terpstra et al. [35]. Cholesterol concentration in lipoprotein fractions were determined on an automatic analyzer (Hitachi 912, Roche Diagnostics GmbH, Mannheim, Germany) using a commercially available kit (test kit # 14899232, Roche Diagnostics GmbH, Mannheim, Germany).

### Statistical analysis

Data are expressed as mean±SD. The data were analysed by non-parametric two or three-way ANOVA with post-hoc Tukey-type multiple comparisons test for effects showing statistical significance in the overall ANOVA The significance level was set to 0.05. The statistical analyses were performed in SAS version 9.2 (SAS Institute Inc., Cary, NC, USA).

## Results

Induction of pulmonary acute phase response was assessed by quantifying mRNA levels of the acute phase gene *Saa3* as a marker of acute phase response, as we have previously found that *Saa3* is the most differentially expressed gene following pulmonary exposure to nanoparticles [19], [25].

### Intratracheal instillation of nanomaterials

Pulmonary induction of the acute phase response was assessed for five different nanomaterials after a single intratracheal instillation (Study 1, Table 1). These nanomaterials were NanoTiO_2_ and NanoCB, a multiwalled carbon nanotube (MWCNT), and two SWCNT (SWCNT_1_, SWCNT_2_). Groups of 6 mice were exposed to doses of 18, 54 and 162 µg/animal and the mice were killed 1, 3 and 28 days after instillation. Twenty-two mice (controls) were instilled with the vehicle (10% mouse broncheoalveolar lavage fluid (BALF) in 0.9% NaCl) at each time point. Some of the results from NanoCB-instilled mice were published previously [20], [21]. Results from NanoTiO_2_-instilled mice were also published previously [26], but *Saa3* mRNA levels were determined independently for the present study to ensure comparability between exposures.

### Saa3 mRNA expression

All particle exposures increased pulmonary *Saa3* mRNA expression in a time- and dose-dependent manner (Table 2). For all particles, the strongest response was seen at the early time points. The strongest response, a 600-fold increase in *Saa3* expression was observed 3 days after exposure to MWCNT. The highest dose of NanoTiO_2_ resulted in a 400-fold increase in *Saa3* mRNA expression in lung at day 1. At day 1, all instilled nanoparticles except for SWCNT_1_ increased mRNA expression of *Saa3* 100-fold or more. This may be compared to a 100-fold induction observed in liver after intraperitoneal injection of lipopolysaccharide [24]. *Saa3* expression was still significantly increased 28 days after exposure for all mice exposed to the highest dose of all the tested materials and for mice exposed to even the lowest dose of MWCNT. This indicates a long-lasting induction of acute phase response.

**Table 2:** Normalised *Saa3* mRNA levels in lung 1, 3 and 28 days after pulmonary exposure to nanomaterials by instillation (study 1).

Particle	Dose	Day1	Day3	Day28
Control	0µg	26±27	23±29	22±27
NanoTiO_2_	18 µg	47±64	25±13	23±7
	54 µg	2254±1625	59±39*	40±28
	162 µg	9585±2529^‡^	447±415^‡^	122±98^‡^
NanoCB	18 µg	1631±1961^‡^	190±206^‡^	25±16
	54 µg	6152±3580^‡^	546±135^‡^	109±96^‡^
	162 µg	7635±2900^‡,§^	1176±312^‡,§^	487±436^‡,§^
MWCNT	18 µg	1343±1224^‡^	903±947^‡^	173±78^‡^
	54 µg	3928±1946^‡^	3496±4159^‡^	643±588^‡^
	162 µg	2459±1503^‡^	14071±9402^‡^	1946±1031^‡^
SWCNT_1_	18 µg	1318±960^‡^	137±88^‡^	50±51
	54 µg	1454±745^‡^	306±195^‡^	143±160^‡^
	162 µg	1616±1042^‡^	627±398^‡^	243±258^‡^
SWCNT_2_	18 µg	2431±2280^‡^	50±13^†^	41±37
	54 µg	9853±11063^‡^	233±186^‡^	105±58^‡^
	162 µg	3736±1886^‡^	473±428^‡^	395±331^‡^

*Saa3* mRNA levels were normalised to 18S and multiplied by 10^7^. Mean±SD is shown.

* p<0.05, †p<0.01, ‡p<0.001, §Data published previously [21].

**Figure 2:**
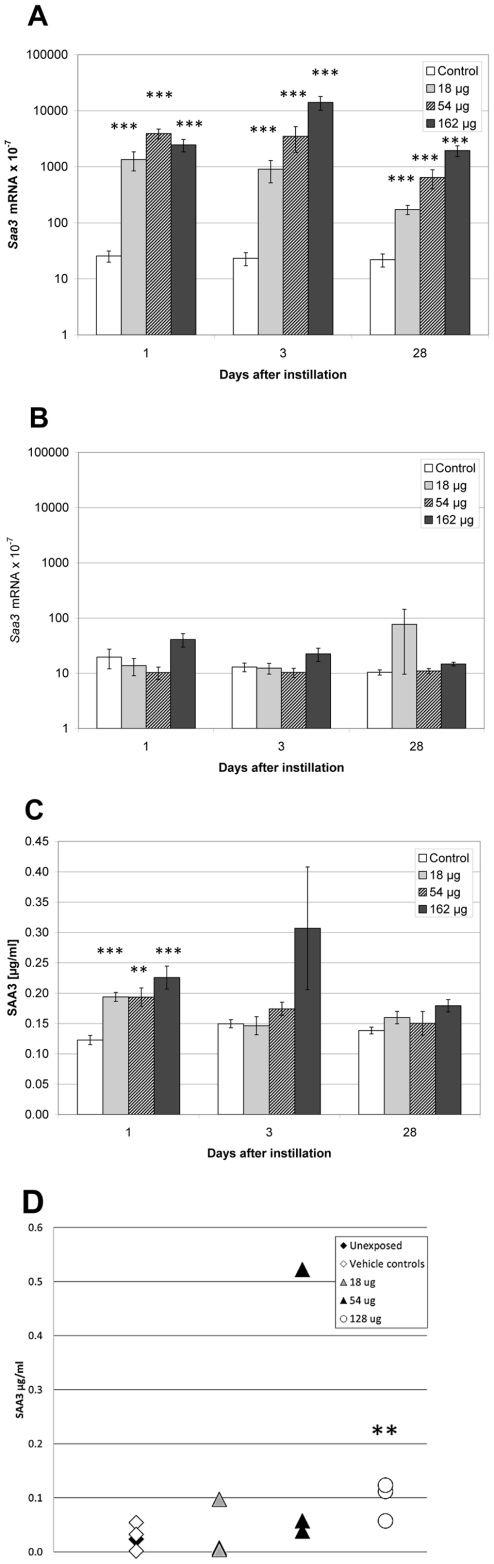
Dose-response effects in mice 1, 3 and 28 days after intratracheal instillation of MWCNT. A) Pulmonary *Saa3* mRNA expression level; B) Hepatic *Saa3* mRNA expression level, C) SAA3 concentration in BALF, and D) SAA3 protein in plasma. *, **, ***: Statistically significant compared to control mice at the 0.5, 0.01 and 0.001 level, respectively.

MWCNT induced the strongest acute phase response in lung, and mRNA expression of *Saa3* was therefore also assessed in liver. MWCNT exposure did not change *Saa3* expression in liver at any dose or time point (Figure 2B).

#### SAA3 protein levels in BALF and plasma

We determined the SAA3 protein levels in BALF by ELISA in mice exposed to MWCNT, because this material strongly induced pulmonary but not the hepatic *Saa3* expression.

The day after exposure, SAA3 protein levels were higher in BALF from MWCNT instilled animals at all doses compared to vehicle instilled controls. No dose-response relationship was seen (Figure 2C). At day 3 and day 28, no statistically significant differences in SAA3 protein levels were detected in BAL.

We determined SAA3 protein in plasma from groups of 3 mice, 3 days after instillation of a single dose of 18 µg, 54 µg, or 128 µg of MWCNT in a separate study (Study 2). Two control groups of unexposed and vehicle exposed animals had similar plasma SAA3 levels. The highest dose of MWCNT instillation increased plasma levels of SAA3 (p<0.01). One mouse instilled with 54 µg presented with a very high level of SAA3 and was considered an outlier. If the outlier was excluded, a dose-dependent increase in plasma SAA3 levels was observed. Without the outlier, plasma levels of SAA3 were 1.4-fold (p>0.05), 1.9-fold (p>0.05) and 3.8-fold (p<0.01) higher in mice exposed to 18, 54 and 128 µg MWCNT, respectively, compared to vehicle instilled and unexposed controls (Figure 2D). The used anti SAA-3 antibody was raised against full length SAA-3. The cross-reactivity with SAA1 and SAA2 is unknown (Millipore, personal communication). The true difference in SAA3 level may therefore be larger if there is cross-reactivity to constitutively expressed SAA isoforms.

#### HDL, LDL and VLDL levels

SAA circulates in blood as a component of HDL, and the classic acute phase response is accompanied by a decrease in HDL-cholesterol [36]. We determined the concentrations of cholesterol in HDL, very low density lipoprotein (VLDL), and low density lipoprotein (LDL) in plasma from mice exposed to MWCNT (Study 2). The concentration of cholesterol in HDL in plasma from MWCNT exposed mice was decreased to ca. 50% at all doses (but not statistically significantly) compared to the HDL-cholesterol concentration of un-exposed and vehicle-exposed mice. The concentrations of cholesterol in VLDL and LDL were unaffected by exposure (results not shown).

The results indicate that pulmonary exposure to these nanomaterials induces pulmonary acute phase response that leads to systemic circulation of the acute phase protein SAA3. Pulmonary acute phase response may be a general response to pulmonary deposition of particles. We therefore also assessed pulmonary acute phase response in mice exposed to NanoTiO_2_, NanoCB and DEP by inhalation and other types of particles by pulmonary instillation.

### Nose-only inhalation of particles

*Saa3* mRNA levels were determined in lungs of mice exposed to 20 mg/m^3^ to DEP or NanoCB for 90 min for 4 consecutive days (Study 3). One hour after the last exposure *Saa3* mRNA levels were 4.4-fold higher after exposure to NanoCB and 17.4-fold increased after exposure to DEP compared to control mice exposed to filtered air (p<0.001, Table 3). We have previously reported that in these same mice, hepatic mRNA expression of *Saa3*, *Saa1*, and *Serum Amyloid P* was unaffected by exposure [24].

**Table 3:** Relative *Saa3* mRNA levels in lung and liver tissue after pulmonary deposition of particles.

Particle	Study	Exposure set-up	Reference	Liver		Lung	
				Control	Exposed	Control	Exposed
NanoCB	3	Inhalation: 4×1.5 h×20 mg/m^3^	[32]	25±37	19±7.8	25±30	111±63*
DEP	3	Inhalation: 4×1.5 h×20 mg/m^3^	[32]	25±37	38±16	25±30	435±662*
Boiler dust	6	Intratracheal instillation: 4×54 µg	[31]	ND†	ND†	563±540	3337±3006*
Straw dust	6	Intratracheal instillation: 4×54 µg	[31]	ND†	ND†	563±540	6389‡5520*

*Saa3* mRNA levels were normalised to 18S and multiplied by 10^7^. Mean±SD is shown. * p<0.001 compared to controls. ^†^ Not determined.

Mice were exposed to 42 mg/m^3^ UV-Titan L181 (NanoTiO_2_) by whole body inhalation 1 h/day for 11 days (Study 4) [23]. Mice were killed 5 and 26–27 days after the last exposure. Pulmonary *Saa3* mRNA was increased 24-fold after 5 days (p<0.001) and 2.1-fold after 26–27 days (p<0.001) compared to controls exposed to filtered air. *Saa3* mRNA levels in the liver were unaffected by exposure (Table 4). We found no difference in *Saa1* or *Sap* mRNA expression in the liver at either time point and the pulmonary expression was below detection level (data not shown).

**Table 4:** Relative *Saa3* mRNA levels in lung and liver tissue after pulmonary deposition of particles by inhalation.

Particle	Study	Exposure set-up	Reference	4–5 days after exposure				∼4weeks after exposure			
				Liver		Lung		Liver		Lung	
				Control	Exposed	Control	Exposed	Control	Exposed	Control	Exposed
NanoTiO_2_	4	11××1 h×42 mg/m^3^	[23]	201±336	70±51	24±12	570±418†	605±369	486±283	1576±4329	3337±3808‡
										(330±376)§	(2384±1722)§
NanoCB	5	11×1 h×42 mg/m^3^	[22]	52±27	116±48	15±8	43±16†	44±33	79±47*	15±6	43±16†

*Saa3* mRNA levels were normalised to 18S and multiplied by 10^7^. Mean±SD is shown. ^*^ p<0.05, ^†^p<0.01, ^‡^p<0.001, ^§^One outlier has been removed in each group.

In a similar exposure set-up mice were exposed to 42 mg/m^3^ NanoCB 1 h/day for 11 consecutive days, also by whole body inhalation (Study 5) [22]. Exposed mice expressed 2.9-fold more pulmonary *Saa3* mRNA (p<0.001) than controls exposed to filtered air five days after termination of exposure, and 1.9-fold (p<0.01) more after 24–25 days. At this time point, *Saa3* mRNA was also increased 1.8-fold (p<0.05) in the liver (Table 4).

Thus, inhalation of particles also induced pulmonary acute phase response that was detectable one month after exposure.

### Intratracheal instillation of particles from a biofuel plant

Pulmonary *Saa3* expression was also determined in mice instilled with dust collected at a biofuel plant (Study 6) [31]. Mice were instilled intratracheally with airborne dust collected at a biofuel plant in the straw storage hall and in the boiler room. Endpoints were determined 1 h after the last instillation. Each mouse was instilled four times with 54 μg dust on four consecutive days. Control mice were similarly instilled with vehicle (0.9% NaCl). Instillation of dust increased expression of *Saa3* statistically significantly, 6-fold for storage hall dust and 11-fold for boiler room dust (Table 3).

### Correlation between pulmonary *Saa3* expression and neutrophil influx

**Figure 3:**
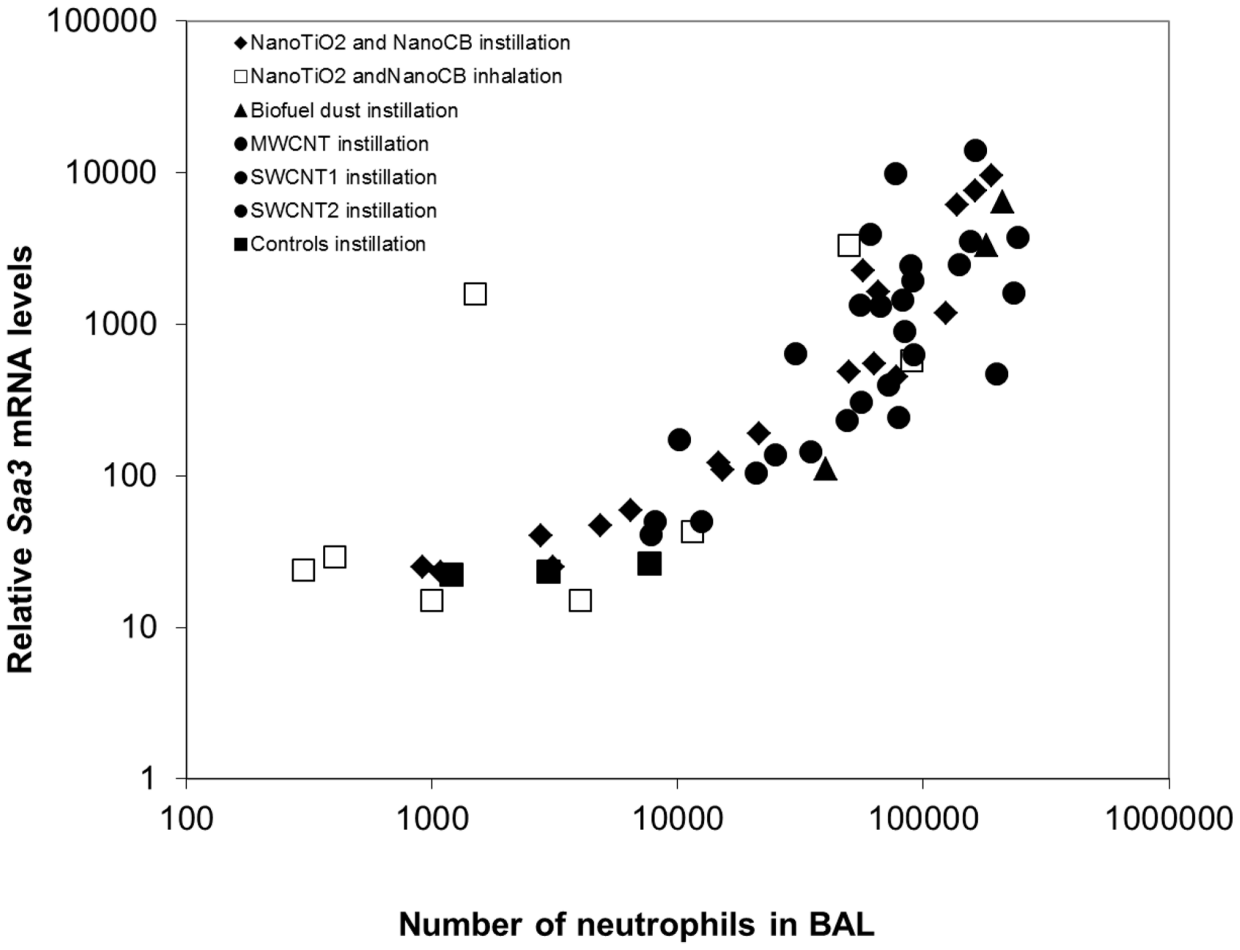
The correlation between the pulmonary mRNA expression of *Saa3* and the influx of neutrophils. The mRNA levels of *Saa3* correlated closely with the number of neutrophils across exposure type, dose, time after exposure and particle type.

SAA is known to be a neutrophil chemoattractant [37]. In all studies, neutrophil levels in BALF were assessed [20], [22], [23], [31], [32], enabling analysis of the correlation between *Saa3* expression levels and the number of neutrophil cell. We found a robust correlation across particle type, method of administration, dose, and time after exposure (Figure 3).

## Discussion

We show that pulmonary exposure to a variety of nanomaterials and other particles results in a rapid and long lasting increase of *Saa3* mRNA levels in lung tissue. This was accompanied by elevated SAA3 protein levels in BAL fluid and in plasma in MWCNT exposed mice. Pulmonary *Saa3* expression co-varied with neutrophil influx in lung lining fluid across particle types, dosing regimens, doses and time points. Only small changes in hepatic expression of *Saa3* were observed. This indicates that pulmonary deposition of particulate matter, including nanoparticles, induces a pulmonary acute phase response. The pulmonary acute phase response correlated closely with neutrophil influx in lung lining fluid.

It is a generally held view that pulmonary inflammation and release of cytokines into the circulation result in a hepatic acute phase response. In contrast, we found a stronger acute phase response in lung than in liver. For most exposures, we only detected increased expression of acute phase protein in lung tissue, whereas for NanoCB, we have previously reported increased expression of the acute phase proteins *Saa3*, *Orm3* and *Saa1* in liver tissue 24 hours after instillation of 162 ug NanoCB/mouse and increased expression of *Crp* 28 after exposure [25]. We also found increased *Saa3* expression in liver 28 days after inhalation exposure to NanoCB (Table 4). However, we consistently found the strongest acute phase response in lung tissue, both in terms of the observed induction fold and in terms of number of differentially expressed acute phase genes.

We used *Saa3* mRNA levels as a biomarker of a pulmonary acute phase response. We have previously found increased expression up to 43 acute phase genes in lung tissue in analyses of global gene expression following pulmonary exposure to NanoCB and NanoTiO_2_ [19], [25], [26]. Signal molecules like cytokines and acute phase proteins are regulated at the level of transcription and the proteins subsequently go into systemic circulation. Therefore increased mRNA expression is the best evidence of the origin of an acute phase response. However, in addition to the increased levels of SAA3 in BAL fluid reported here, we have previously reported that the protein levels of two acute phase protein, granulocyte colony-stimulating factor (G-CSF) and granulocyte-macrophage colony-stimulating-factor (GM-CSF) were increased 6-fold and 2.5-fold, respectively, in lung tissue following instillation of NanoTiO_2_ [26] and 3-fold and 1.3-fold increased, respectively, following instillation of NanoCB [38]. In both cases, G-CSF protein levels increased in a dose-dependent manner with increasing exposure dose. Moreover, dose-dependently increased protein levels of IL-1β, IL-6, and CXCL1 were also found in lung tissue 28 days following instillation of NanoCB [38]. In addition, Teeguarden et al [39] exposed mice to different kinds of nanoparticles twice a week for three weeks to a total dose of 240 µg by aspiration. Twenty-four hours after last exposure the protein levels of the acute-phase proteins C3, Fn1, S100A8 and S100A9 were increased in the lung tissue in mice exposed to single-walled carbon nanotubes. Moreover, the level of the acute-phase protein haptoglobin was increased in BALF collected 24 h after a single intratracheal instillation of 200 µg NanoCB/mouse [40].

CRP levels is a risk factor for cardiovascular disease [41]. It is debated whether plasma levels of CRP and SAA are just passive bystanders of disease or whether these acute phase proteins are causally related to cardiovascular disease [42]. Genetic variations and haplotypes in the CRP gene are associated with differences in plasma CRP levels but not with the risk of coronary heart disease [14], [43]. This suggests that CRP levels in plasma are not causally related to coronary heart disease [44], [45] despite their prospective association to risk of CHD in prospective studies [46]. Instead, evidence suggests a causative role of SAA: In mice, SAA promotes atherosclerosis directly [47], impairs endothelial dysfunction [30] and is a chemoattractant of monocytes and neutrophils [37], [48]. Strong inducers of SAA such as lipopolysaccharide [49] as well as oral infection with periodontal pathogens accelerate the development of atherosclerotic plaques both in APOE^−/−^ mice [50] and in APOE^+/−^ heterozygotes [51]. It was recently demonstrated that Lenti-virus mediated overexpression of SAA1 in APOE −/− mice led to accelerated plaque progression. This indicates that SAA1 is causal in plaque progression and thus leads to atherosclerosis [47]. Plaque progression may be caused by inhibition of reverse cholesterol transport from macrophages to the liver [36]. During acute phase response, SAA proteins are incorporated into HDL substituting apoA-I. The acute phase HDL is less able to facilitate reverse cholesterol transport from macrophages to faeces [52], [53] and promotes the formation of foam cells and subsequent plaque progression. In line with this, adenovirus mediated overexpression of murine SAA decreased macrophage-to-faeces reverse cholesterol transport in vivo in mice [52]. SAA3 is a pseudogene in humans. However, we found a close correlation between *Saa1* and *Saa3* expression in murine lung tissue, and chose *Saa3* as the biomarker because of the larger dynamic range in gene expression.

We here report a close correlation between *Saa3* gene expression and neutrophil influx (Figure 3). It has previously been shown that neutrophil influx in response to particle exposure correlates with the total surface area of the deposited particles [33], [54]. Thus, we have indirectly established a link between total inhaled surface area of particles and acute phase response and thus cardiovascular risk.

Plaque progression in APOE −/− mice has been used as an animal model of cardiovascular disease. Interestingly, inhalation of diesel exhaust particles [55], aspiration of single walled carbon nanotubes [27] and pulmonary instillation of TiO_2_ nanoparticles [29] lead to increased plaque progression in APOE −/− mice. However, these studies were long lasting, used large groups of transgenic mice and had a small dynamic range. They allowed for demonstration of effect but did not allow for comparison of atherosclerotic effect of different particles. The use of biomarkers of the acute phase response has been complicated by the fact that inhalation of particles was not accompanied by a hepatic acute phase response [24]. The presented results indicate that pulmonary *Saa3* expression may be used as a biomarker that allows for comparison of the cardiovascular toxicity of different particles and nanomaterials in wildtype animal models. Furthermore, the large dynamic range increases sensitivity, thus reducing the number of animals needed to detect an effect.

In the mice inhaling DEP and NanoCB for 90 min for 4 consecutive days (Study 3), pulmonary expression of *Interleukin-6* (*Il-6*)and *Tumour necrosis factor* (*Tnf*) was increased, but to a similar degree by NanoCB and DEP while *Interleukin-1 beta* (*Il1β*) mRNA expression was low and unaffected by exposure [32]. However, the influx of neutrophils was very different for the two exposures. In DEP exposed mice, the percentage of neutrophils in BALF cells was increased from 4% in controls to 15% in DEP exposed, whereas no increase in neutrophils was observed in mice exposed to NanoCB [32]. Pulmonary *Saa3* gene expression covaried with the neutrophil influx, since *Saa3* expression was 4 times higher in DEP exposed mice than in NanoCB exposed mice. Because *Il-1β, Il6* and *Tnf* expression levels were comparable for the two exposures, it appears as if the neutrophil influx is more closely associated with *Saa3* expression levels. SAA is a chemoattractant for neutrophils, and the chemoattractant effect of SAA is blocked when SAA forms complexes with HDL [37]. It has previously been demonstrated that macrophages excrete SAA [56]. We suggest that nanoparticles stimulate macrophages to secrete SAA which acts as an important chemoattractant for neutrophils. This would link pulmonary particle exposure directly with cardiovascular disease. Many other cytokines are chemoattractants for neutrophils [57].

In controlled human exposure studies, exposure to ambient particulate matter increased blood levels of the acute phase protein CRP [58]. Ambient PM(2.5) has been associated with increased blood levels of CRP [59]–[62], and blood levels of SAA were increased in volunteers exposed to wood smoke [63]. In a cross-sectional study of serum levels of CRP and SAA, LDL and triglyceride levels were positively associated with CRP levels and HDL levels correlated negatively with CRP, although the latter association was not statistically significant. No associations between serum levels of SAA and HDL, LDL or triglycerides were observed [64]. When humans undergo acute phase response, LDL synthesis is increased, but LDL levels in blood decrease due to up-regulation of LDL receptor activity [65]. HDL blood levels decrease, and blood levels of triglycerides increase [65]. Patients with acute myocardial infarction also undergo an acute phase response. Cholesterol biosynthesis was assessed in 34 patients hospitalised with acute myocardial infarction and cholesterol biosynthesis was found to be 23 and 29% increased 1 and 2 days after hospitalisation, respectively [66]. Thus, the observed increased cholesterol content in liver and lowered serum HDL levels observed in mice following pulmonary exposure to NanoCB [25] is similar to what is observed in humans.

Given the close association between acute phase response and risk of cardiovascular disease, our results indicate that exposures to NanoCB and NanoTiO_2_ at doses which are comparable to 3 or 14 working days at the current Danish occupational exposure limits of 3.5 mg/m^3^ for CB and 9.75 mg/m^3^ for TiO_2_, respectively [20], [21] lead to increased *Saa3* expression even 28 days after last exposure. Similar results were obtained after 11 days inhalation of NanoCB and NanoTiO_2_ at doses corresponding to 1.5 or 0.5 times the current Danish occupational exposure limits. Also dust collected in a biofuel facility with high exposure to both particles and endotoxins increased *Saa3* levels in lungs of exposed mice [31]. Human exposure levels at this plant were 0.62 mg/m^3^ dust and 1298 EU/m^3^ endotoxin in the storage hall and 1.18 mg/m^3^ dust and 2178 EU/m^3^ endotoxin in the boiler room [31]. The 4 times 54 µg µg/animal administered to the animals over 4 days corresponded to 2 weeks of exposure [31].

## Conclusions

Pulmonary exposure to several nanomaterials and other particles led to a pulmonary acute phase response characterised by long-lasting increased expression of *Saa3* mRNA and plasma SAA3 protein. SAA has a key role in promotion of plaque progression. We therefore propose that the pulmonary acute phase response may constitute a causative link between particle inhalation and risk of cardiovascular disease. The SAA is a potent chemotactic factor for neutrophils and the mRNA levels of *Saa3* correlated closely with the number of neutrophils in BAL fluid across exposure type, dose and time after exposure and particle type. Our results suggest that nanoparticles differ in their ability to induce acute phase response and therefore that evaluation of the potency to induce pulmonary acute phase response may allow for the rating of different nanoparticles in relation to risk of cardiovascular disease.

## References

[1] Pope CA III, Burnett RT, Thurston GD, Thun MJ, Calle EE, et al (2004) Cardiovascular mortality and long-term exposure to particulate air pollution: epidemiological evidence of general pathophysiological pathways of disease. Circulation 109: 71–77. 14676145 10.1161/01.CIR.0000108927.80044.7F

[2] Fang SC, Cassidy A, Christiani DC (2010) A Systematic Review of Occupational Exposure to Particulate Matter and Cardiovascular Disease. International Journal of Environmental Research and Public Health 7: 1773–1806. 20617059 10.3390/ijerph7041773 PMC2872342

[3] Oberdorster G, Oberdorster E, Oberdorster J (2005) Nanotoxicology: an emerging discipline evolving from studies of ultrafine particles. Environ Health Perspect 113: 823–839. 16002369 10.1289/ehp.7339 PMC1257642

[4] Taubes G (2002) Cardiovascular disease. Does inflammation cut to the heart of the matter? Science 296: 242–245. 11951014 10.1126/science.296.5566.242

[5] Packard RR, Libby P (2008) Inflammation in atherosclerosis: from vascular biology to biomarker discovery and risk prediction. Clin Chem 54: 24–38. 18160725 10.1373/clinchem.2007.097360

[6] Gabay C, Kushner I (1999) Mechanisms of disease: Acute-phase proteins and other systemic responses to inflammation. New England Journal of Medicine 340: 448–454. 9971870 10.1056/NEJM199902113400607

[7] Lowe GD (2001) The relationship between infection, inflammation, and cardiovascular disease: an overview. Ann Periodontol 6: 1–8. 10.1902/annals.2001.6.1.1 11887452

[8] Mezaki T, Matsubara T, Hori T, Higuchi K, Nakamura A, et al (2003) Plasma levels of soluble thrombomodulin, C-reactive protein, and serum amyloid A protein in the atherosclerotic coronary circulation. Jpn Heart J 44: 601–612. 14587642 10.1536/jhj.44.601

[9] Libby P, Okamoto Y, Rocha VZ, Folco E (2010) Inflammation in atherosclerosis: transition from theory to practice. Circ J 74: 213–220. 20065609 10.1253/circj.cj-09-0706

[10] Pussinen PJ, Tuomisto K, Jousilahti P, Havulinna AS, Sundvall J, et al (2007) Endotoxemia, immune response to periodontal pathogens, and systemic inflammation associate with incident cardiovascular disease events. Arteriosclerosis Thrombosis and Vascular Biology 27: 1433–1439. 10.1161/ATVBAHA.106.138743 17363692

[11] Estabragh ZR, Mamas MA (2013) The cardiovascular manifestations of influenza: A systematic review. Int J Cardiol. 10.1016/j.ijcard.2013.01.274 23474244

[12] Ridker PM, Hennekens CH, Buring JE, Rifai N (2000) C-reactive protein and other markers of inflammation in the prediction of cardiovascular disease in women. N Engl J Med 342: 836–843. 10733371 10.1056/NEJM200003233421202

[13] Hertel S, Viehmann A, Moebus S, Mann K, Brocker-Preuss M, et al (2010) Influence of short-term exposure to ultrafine and fine particles on systemic inflammation. European Journal of Epidemiology 25: 581–592. 20559688 10.1007/s10654-010-9477-x

[14] Pai JK, Pischon T, Ma J, Manson JE, Hankinson SE, et al (2004) Inflammatory markers and the risk of coronary heart disease in men and women. N Engl J Med 351: 2599–2610. 15602020 10.1056/NEJMoa040967

[15] Uhlar CM, Whitehead AS (1999) Serum amyloid A, the major vertebrate acute-phase reactant. European Journal of Biochemistry 265: 501–523. 10504381 10.1046/j.1432-1327.1999.00657.x

[16] Chiba T, Han CY, Vaisar T, Shimokado K, Kargi A, et al (2009) Serum amyloid A3 does not contribute to circulating SAA levels. Journal of Lipid Research 50: 1353–1362. 19286646 10.1194/jlr.M900089-JLR200 PMC2694334

[17] Meek RL, Urielishoval S, Benditt EP (1994) Expression of Apolipoprotein Serum Amyloid-A Messenger-Rna in Human Atherosclerotic Lesions and Cultured Vascular Cells - Implications for Serum Amyloid-A Function. Proceedings of the National Academy of Sciences of the United States of America 91: 3186–3190. 8159722 10.1073/pnas.91.8.3186 PMC43540

[18] Whitehead AS, Zahedi K, Rits M, Mortensen RF, Lelias JM (1990) Mouse C-reactive protein. Generation of cDNA clones, structural analysis, and induction of mRNA during inflammation. Biochem J 266: 283–290. 2310378 10.1042/bj2660283 PMC1131125

[19] Halappanavar S, Jackson P, Williams A, Jensen KA, Hougaard KS, et al (2011) Pulmonary response to surface-coated nanotitanium dioxide particles includes induction of acute phase response genes, inflammatory cascades, and changes in microRNAs: A toxicogenomic study. Environ Mol Mutagen 52: 425–439. 21259345 10.1002/em.20639 PMC3210826

[20] Saber AT, Jacobsen NR, Mortensen A, Szarek J, Jackson P, et al (2012) Nanotitanium dioxide toxicity in mouse lung is reduced in sanding dust from paint. Part Fibre Toxicol 9: 4. 22300483 10.1186/1743-8977-9-4 PMC3298479

[21] Bourdon JA, Saber AT, Jacobsen NR, Jensen KA, Madsen AM, et al (2012) Carbon black nanoparticle instillation induces sustained inflammation and genotoxicity in mouse lung and liver. Part Fibre Toxicol 9: 5. 22300514 10.1186/1743-8977-9-5 PMC3293019

[22] Jackson P, Hougaard KS, Boisen AM, Jacobsen NR, Jensen KA, et al (2012) Pulmonary exposure to carbon black by inhalation or instillation in pregnant mice: effects on liver DNA strand breaks in dams and offspring. Nanotoxicology 6: 486–500. 21649560 10.3109/17435390.2011.587902 PMC3411122

[23] Hougaard KS, Jackson P, Jensen KA, Sloth JJ, Loschner K, et al (2010) Effects of prenatal exposure to surface-coated nanosized titanium dioxide (UV-Titan). A study in mice. Part Fibre Toxicol 7: 16. 20546558 10.1186/1743-8977-7-16 PMC2908059

[24] Saber AT, Halappanavar S, Folkmann JK, Bornholdt J, Boisen AM, et al (2009) Lack of acute phase response in the livers of mice exposed to diesel exhaust particles or carbon black by inhalation. Part Fibre Toxicol 6: 12. 19374780 10.1186/1743-8977-6-12 PMC2673201

[25] Bourdon JA, Halappanavar S, Saber AT, Jacobsen NR, Williams A, et al (2012) Hepatic and pulmonary toxicogenomic profiles in mice intratracheally instilled with carbon black nanoparticles reveal pulmonary inflammation, acute phase response, and alterations in lipid homeostasis. Toxicol Sci 127: 474–484. 22461453 10.1093/toxsci/kfs119 PMC3355316

[26] Husain M, Saber AT, Guo C, Jacobsen NR, Jensen KA, et al (2013) Pulmonary instillation of low doses of titanium dioxide nanoparticles in mice leads to particle retention and gene expression changes in the absence of inflammation. Toxicol Appl Pharmacol 269: 250–262. 23557971 10.1016/j.taap.2013.03.018

[27] Li Z, Hulderman T, Salmen R, Chapman R, Leonard SS, et al (2007) Cardiovascular effects of pulmonary exposure to single-wall carbon nanotubes. Environmental Health Perspectives 115: 377–382. 17431486 10.1289/ehp.9688 PMC1849906

[28] Chen LC, Nadziejko C (2005) Effects of subchronic exposures to concentrated ambient particles (CAPs) in mice: V. CAPs exacerbate aortic plaque development in hyperlipidemic mice. Inhalation Toxicology 17: 217–224. 15804939 10.1080/08958370590912815

[29] Mikkelsen L, Sheykhzade M, Jensen KA, Saber AT, Jacobsen NR, et al (2011) Modest effect on plaque progression and vasodilatory function in atherosclerosis-prone mice exposed to nanosized TiO2. Part Fibre Toxicol 8: 32. 22074227 10.1186/1743-8977-8-32 PMC3245428

[30] Wang X, Chai H, Wang Z, Lin PH, Yao Q, et al (2008) Serum amyloid A induces endothelial dysfunction in porcine coronary arteries and human coronary artery endothelial cells. Am J Physiol Heart Circ Physiol 295: H2399–H2408. 18931033 10.1152/ajpheart.00238.2008 PMC2614654

[31] Madsen AM, Saber AT, Nordly P, Sharma AK, Wallin H, et al (2008) Inflammation but no DNA (deoxyribonucleic acid) damage in mice exposed to airborne dust from a biofuel plant. Scand J Work Environ Health 34: 278–7. 18820821 10.5271/sjweh.1272

[32] Saber AT, Bornholdt J, Dybdahl M, Sharma AK, Loft S, et al (2005) Tumor necrosis factor is not required for particle-induced genotoxicity and pulmonary inflammation. Arch Toxicol 79: 177–182. 15798890 10.1007/s00204-004-0613-9

[33] Saber AT, Jensen KA, Jacobsen NR, Birkedal R, Mikkelsen L, et al (2012) Inflammatory and genotoxic effects of nanoparticles designed for inclusion in paints and lacquers. Nanotoxicology 6: 453–471. 21649461 10.3109/17435390.2011.587900

[34] Livak KJ, Schmittgen TD (2001) Analysis of relative gene expression data using real-time quantitative PCR and the 2(-Delta Delta C(T)) Method. Methods 25: 402–408. 11846609 10.1006/meth.2001.1262

[35] Terpstra AH, Woodward CJ, Sanchez-Muniz FJ (1981) Improved techniques for the separation of serum lipoproteins by density gradient ultracentrifugation: visualization by prestaining and rapid separation of serum lipoproteins from small volumes of serum. Anal Biochem 111: 149–157. 6165257 10.1016/0003-2697(81)90243-8

[36] Feingold KR, Grunfeld C (2010) The acute phase response inhibits reverse cholesterol transport. Journal of Lipid Research 51: 682–684. 20071695 10.1194/jlr.E005454 PMC2842157

[37] Badolato R, Wang JM, Murphy WJ, Lloyd AR, Michiel DF, et al (1994) Serum amyloid A is a chemoattractant: induction of migration, adhesion, and tissue infiltration of monocytes and polymorphonuclear leukocytes. J Exp Med 180: 203–209. 7516407 10.1084/jem.180.1.203 PMC2191543

[38] Jackson P, Hougaard KS, Vogel U, Wu D, Casavant L, et al (2012) Exposure of pregnant mice to carbon black by intratracheal instillation: toxicogenomic effects in dams and offspring. Mutat Res 745: 73–83. 22001195 10.1016/j.mrgentox.2011.09.018

[39] Teeguarden JG, Webb-Robertson BJ, Waters KM, Murray AR, Kisin ER, et al (2011) Comparative proteomics and pulmonary toxicity of instilled single-walled carbon nanotubes, crocidolite asbestos, and ultrafine carbon black in mice. Toxicol Sci 120: 123–135. 21135415 10.1093/toxsci/kfq363 PMC3044201

[40] Chang CC, Chen SH, Ho SH, Yang CY, Wang HD, et al (2007) Proteomic analysis of proteins from bronchoalveolar lavage fluid reveals the action mechanism of ultrafine carbon black-induced lung injury in mice. Proteomics 7: 4388–4397. 17963277 10.1002/pmic.200700164

[41] Kaptoge S, Di AE, Pennells L, Wood AM, White IR, et al (2012) C-reactive protein, fibrinogen, and cardiovascular disease prediction. N Engl J Med 367: 1310–1320. 23034020 10.1056/NEJMoa1107477 PMC3714101

[42] Stulnig TM (2013) C-reactive protein, fibrinogen, and cardiovascular risk. N Engl J Med 368: 84–85. 10.1056/NEJMc1213688 23281989

[43] Elliott P, Chambers JC, Zhang W, Clarke R, Hopewell JC, et al (2009) Genetic Loci associated with C-reactive protein levels and risk of coronary heart disease. JAMA 302: 37–48. 19567438 10.1001/jama.2009.954 PMC2803020

[44] Vogel U, Jensen MK, Due KM, Rimm EB, Wallin H, et al (2011) The NFKB1 ATTG ins/del polymorphism and risk of coronary heart disease in three independent populations. Atherosclerosis 219: 200–204. 21726863 10.1016/j.atherosclerosis.2011.06.018 PMC3827022

[45] Vogel U (in press) Commentary. Atherosclerosis.

[46] Danesh J, Whincup P, Walker M, Lennon L, Thomson A, et al (2000) Low grade inflammation and coronary heart disease: prospective study and updated meta-analyses. BMJ 321: 199–204. 10903648 10.1136/bmj.321.7255.199 PMC27435

[47] Dong Z, Wu T, Qin W, An C, Wang Z, et al.. (2011) Serum Amyloid A Directly Accelerates the Progression of Atherosclerosis in Apolipoprotein E-Deficient Mice. Mol Med. 10.2119/molmed.2011.00186 PMC3321823 21953420

[48] King VL, Thompson J, Tannock LR (2011) Serum amyloid A in atherosclerosis. Curr Opin Lipidol 22: 302–307. 21734573 10.1097/MOL.0b013e3283488c39

[49] Gitlin JM, Loftin CD (2009) Cyclooxygenase-2 inhibition increases lipopolysaccharide-induced atherosclerosis in mice. Cardiovascular Research 81: 400–407. 18948273 10.1093/cvr/cvn286 PMC2639107

[50] Lalla E, Lamster IB, Hofmann MA, Bucciarelli L, Jerud AP, et al (2003) Oral infection with a periodontal pathogen accelerates early atherosclerosis in apolipoprotein E-null mice. Arteriosclerosis Thrombosis and Vascular Biology 23: 1405–1411. 10.1161/01.ATV.0000082462.26258.FE 12816879

[51] Li L, Messas E, Batista EL, Levine RA, Amar S (2002) Porphyromonas gingivalis infection accelerates the progression of atherosclerosis in a heterozygous apolipoprotein E-deficient murine model. Circulation 105: 861–867. 11854128 10.1161/hc0702.104178

[52] Annema W, Nijstad N, Tolle M, de Boer JF, Buijs RVC, et al (2010) Myeloperoxidase and serum amyloid A contribute to impaired in vivo reverse cholesterol transport during the acute phase response but not group IIA secretory phospholipase A(2). Journal of Lipid Research 51: 743–754. 20061576 10.1194/jlr.M000323 PMC2842154

[53] Jahangiri A, de Beer MC, Noffsinger V, Tannock LR, Ramaiah C, et al (2009) HDL remodeling during the acute phase response. Arterioscler Thromb Vasc Biol 29: 261–267. 19008529 10.1161/ATVBAHA.108.178681 PMC2760005

[54] Duffin R, Tran L, Brown D, Stone V, Donaldson K (2007) Proinflammogenic effects of low-toxicity and metal nanoparticles in vivo and in vitro: highlighting the role of particle surface area and surface reactivity. Inhal Toxicol 19: 849–856. 17687716 10.1080/08958370701479323

[55] Bai N, Kido T, Suzuki H, Yang G, Kavanagh TJ, Kaufman JD, Rosenfeld ME, van BC, Eeden SF (2011) Changes in atherosclerotic plaques induced by inhalation of diesel exhaust. Atherosclerosis. 10.1016/j.atherosclerosis.2011.02.019 PMC4631611 21435644

[56] RL, Eriksen N, Benditt EP (1992) Murine serum amyloid A3 is a high density apolipoprotein and is secreted by macrophages. Proc Natl Acad Sci U S A 89: 7949–7952. 1518819 10.1073/pnas.89.17.7949 PMC49832

[57] A, Wuyts A, Proost P, Struyf S, Opdenakker G, van DJ (1996) Leukocyte migration and activation by murine chemokines. Immunobiology 195: 499–521. 8933154 10.1016/S0171-2985(96)80019-2

[58] Pope CA III, Hansen ML, Long RW, Nielsen KR, Eatough NL, Wilson WE, Eatough DJ (2004) Ambient particulate air pollution, heart rate variability, and blood markers of inflammation in a panel of elderly subjects. Environ Health Perspect 112: 339–345. 14998750 10.1289/ehp.6588 PMC1241864

[59] Riediker M, Cascio WE, Griggs TR, Herbst MC, Bromberg PA, Neas L, Williams RW, Devlin RB (2004) Particulate matter exposure in cars is associated with cardiovascular effects in healthy young men. Am J Respir Crit Care Med 169: 934–940. 14962820 10.1164/rccm.200310-1463OC

[60] Peters A, Frohlich M, Doring A, Immervoll T, Wichmann HE, Hutchinson WL, Pepys MB, Koenig W (2001) Particulate air pollution is associated with an acute phase response in men; results from the MONICA-Augsburg Study. Eur Heart J 22: 1198–1204. 11440492 10.1053/euhj.2000.2483

[61] Delfino RJ, Staimer N, Tjoa T, Polidori A, Arhami M, Gillen DL, Kleinman MT, Vaziri ND, Longhurst J, Zaldivar F, Sioutas C (2008) Circulating biomarkers of inflammation, antioxidant activity, and platelet activation are associated with primary combustion aerosols in subjects with coronary artery disease. Environ Health Perspect 116: 898–906. 18629312 10.1289/ehp.11189 PMC2453158

[62] Huttunen K, Siponen T, Salonen I, Yli-Tuomi T, Aurela M, Dufva H, Hillamo R, Linkola E, Pekkanen J, Pennanen A, Peters A, Salonen RO, Schneider A, Tiittanen P, Hirvonen MR, Lanki T (2012) Low-level exposure to ambient particulate matter is associated with systemic inflammation in ischemic heart disease patients. Environ Res 116: 44–51. 22541720 10.1016/j.envres.2012.04.004

[63] Barregard L, Sallsten G, Gustafson P, Andersson L, Johansson L, Basu S, Stigendal L (2006) Experimental exposure to wood-smoke particles in healthy humans: effects on markers of inflammation, coagulation, and lipid peroxidation. Inhal Toxicol 18: 845–853. 16864402 10.1080/08958370600685798

[64] Danesh J, Muir J, Wong YK, Ward M, Gallimore JR, Pepys MB (1999) Risk factors for coronary heart disease and acute-phase proteins. A population-based study. Eur Heart J 20: 954–959. 10361047 10.1053/euhj.1998.1309

[65] Balci B (2011) The modification of serum lipids after acute coronary syndrome and importance in clinical practice. Curr Cardiol Rev 7: 272–276. 22758629 10.2174/157340311799960690 PMC3322446

[66] Pfohl M, Schreiber I, Liebich HM, Haring HU, Hoffmeister HM (1999) Upregulation of cholesterol synthesis after acute myocardial infarction–is cholesterol a positive acute phase reactant? Atherosclerosis 142: 389–393. 10030390 10.1016/s0021-9150(98)00242-1

